# *SATB2*-Associated Syndrome Due to a c.715C>T:p(Arg239*) Variant in Adulthood: Natural History and Literature Review

**DOI:** 10.3390/genes14040882

**Published:** 2023-04-08

**Authors:** Matheus de Mello Copelli, Eleonore Pairet, Milena Atique-Tacla, Társis Paiva Vieira, Simone Appenzeller, Raphaël Helaers, Miikka Vikkula, Vera Lúcia Gil-da-Silva-Lopes

**Affiliations:** 1 Department of Translational Medicine, Area of Medical Genetics and Genomic Medicine, University of Campinas (UNICAMP), Campinas CEP 13083-887, SP, Brazil; m159476@dac.unicamp.br (M.d.M.C.);; 2 Human Molecular Genetics, de Duve Institute, Université Catholique de Louvain, 1200 Brussels, Belgium; 3 Department of Orthopedics, Rheumatology and Traumatology, School of Medical Science, University of Campinas (UNICAMP), Campinas CEP 13083-887, SP, Brazil

**Keywords:** natural history, whole-exome sequencing, cleft palate, behavior problems, osteopenia, *SATB2*

## Abstract

*SATB2*-associated syndrome (SAS) is a rare condition, and it is characterized by severe developmental delay/intellectual disability, especially severe speech delay/or absence, craniofacial abnormalities, and behavioral problems. Most of the published reports are limited to children, with little information about the natural history of the disease and the possible novel signs and symptoms or behavioral changes in adulthood. We describe the management and follow-up of a 25-year-old male with SAS due to a de novo heterozygous nonsense variant *SATB2*:c.715C>T:p.(Arg239*) identified by whole-exome sequencing and review the literature. The case herein described contributes to a better characterization of the natural history of this genetic condition and in addition to the genotype–phenotype correlation of the *SATB2*:c.715C>T:p.(Arg239*) variant in SAS, highlights some particularities of its management.

## 1. Introduction

“The Glass Syndrome” (MIM# 612313) was the first denomination related to alterations in the Special AT-rich Sequence-Binding Protein 2 gene (*SATB2*) after the description of one patient presenting a deletion on chromosome 2q32.2q33.1 [1]. Subsequently, microdeletions, duplications, and translocations encompassing *SATB2* were reported suggesting that haploinsufficiency of this gene might be responsible for the 2q32q33 microdeletion syndrome, mainly characterized by speech delay/or absence, learning difficulties, growth retardation, dysmorphic features, thin/sparse hair, feeding difficulties, and cleft or high palate [2]. Despite the prevalence being unknown, there are two recent studies of individuals with undiagnosed Intellectual Disability/Developmental Delay (ID/DD) that demonstrated a frequency of SAS varying from 0.24 to 0.3% [3,4].

Until 2014, two individuals presenting the same nonsense variant *SATB2*:c.715C>T:p.(Arg239*) in the *SATB2* gene were reported [5,6]. A description of small duplications and four deletions restricted to the *SATB2* gene were also found [2,7,8,9,10,11,12,13,14]. These reports supported the proposal of a clinical entity termed SAS (*SATB2*-associated syndrome). It is characterized by severe DD/ID, especially severe speech delay/or absence, craniofacial abnormalities, such as micrognathia, dental and palatal abnormalities, behavioral problems, and subtle dysmorphic features [6,15]. Later, skeletal anomalies and osteopenia/osteoporosis were included as part of the phenotype [15,16]. To date, the SATB2 Gene Foundation (https://satb2gene.org, accessed on 15 December 2022) reports more than 650 individuals with this condition.

The *SATB2* gene is mapped on chromosome 2q32-q33, contains 11 exons spanning 191 kb, and encodes a 733-amino acid protein with a high degree of evolutionary conservation [17]. Encoding a cell-type-specific transcription factor, the *SATB2* gene is involved in transcription, chromatin regulation, and repression and activation of specific genes [5,18,19]. In vertebrates, it is expressed in the brain, branchial arch derivates, and sites of bone formation and is also considered a cleft palate candidate gene because of its pattern of expression observed in embryos during palatogenesis [6,17,20,21]. It was suggested that the *SATB2* loss-of-function variant in mice plays a fundamental role in the coordination of jaw development and osteoblast differentiation, implicating its role in the regulation of craniofacial development [20,22].

Haploinsufficiency is assumed as the main causative mechanism for the clinical spectrum seen in human patients with SAS and is the result of small indels as well as large deletions and translocations, which directly disrupt the *SATB2* gene [10,14,20,23]. However, the exact pathophysiology of sequence variants is still unknown. A few studies suggest that there may be dominant-negative effects by which the truncated protein disturbs the repressive function of the wild-type *SATB2* [5,24]. This dominant-negative effect could occur due to the truncated p.(Arg239*) forming a dimer with the wild-type in the nucleus and interfering with its normal activity.

Our case report describes the management and follow-up of a 25-year-old male with SAS due to a de novo heterozygous nonsense variant *SATB2*:c.715C>T:p.(Arg239*).

## 2. Materials and Methods

### 2.1. DNA Sample

Genomic DNA was obtained from peripheral blood samples using phenol/chloroform extraction, according to a standardized protocol, and purified by Microcon-30 kDa Centrifugal Filter Unit with Ultracel-30 membrane (Millipore, Billerica, MA, USA).

### 2.2. Whole-Exome Sequencing (WES)

WES of patient’s sample was performed by Macrogen, Inc.^©^ (Seoul, Republic of Korea). WES was performed using 1µg of genomic DNA, which was fragmented into a library of small segments, and capture was performed using the Agilent SureSelectXT Human All Exon Kit V6 (Agilent Technologies^®^, Santa Clara, CA, USA). Sequencing was performed on Illumina NovaSeq 6000 Sequencing System (Illumina, Inc.^©^, San Diego, CA, USA) to generate paired-end, 2 × 150 bp reads, and with on-target 140× coverage. The raw data were extracted in Fastq format.

### 2.3. Data Analysis

Raw data (fasta files) were aligned to the reference human sequence assembly (GRCh37) using BWA 0.7.15 (Wellcome Trust Sanger Institute, Hinxton, UK) and imported to the bioinformatics computing cluster for data analysis. Variant detection and calling were performed on aligned sequences (bam files) using Picard 1.107 for removal of duplicates and quality value recalibration and GATK 3.3 Haplotype Caller for variant calling (both from the Broad Institute, Cambridge, MA, USA). The variant (vcf) files thus generated were imported into a database and further analyzed using an in-house NGS data analysis framework used for variant annotation, filtering, and visualization. Filtering was retained for variants that satisfied the following criteria: (i) pass GATK standard quality control filters; (ii) within a list of 159 candidate genes for oral clefts; (iii) missense, nonsense, frameshift, and splice-site changes; (iv) <1% allele frequency in the ExAC database of WES from 60,706 unrelated individuals (http://exac.broadinstitute.org/, accessed on 10 December 2022); (v) not detected in samples from individuals with unrelated pathologies (or unaffected controls) in the in-house database of 1800 WES; (vi) for missense variants, predicted to affect protein function by at least 3 out of 6 prediction tools (DAMAGING in Sift, DELETERIOUS in LRT, HIGH or MEDIUM in Mutation Assessor, DAMAGING in FATHMM, and DISEASE CAUSING (AUTOMATIC or not) in Mutation Taster, a score >0.5 in Polyphen2 (hdiv or hvar)).

### 2.4. Validation by Sanger Sequencing

Sanger sequencing was used to validate the variant identified, and segregation analysis was performed according to a standardized protocol. The Ensembl Genome Browser (assembly GRCh37/hg19) was used to design the primers (Forward: 5′-GCTGGAGCTTCCATCGTTGTA-3′/Reverse: 5′-GTTGCCTTACAAGTTGTGGACT-3′). The fragment of interest was amplified by Polymerase Chain Reaction (PCR) and purified with Exo-Sap enzyme (Thermo Fisher Scientific Inc.—Applied Biosystems™, Carlsbad, CA, USA), and the sequencing reaction was performed with BigDye™ Terminator v3.1 Cycle Sequencing Kit (Thermo Fisher Scientific Inc.—Life Technologies, Carlsbad, CA, USA). Subsequently, sequence reading was performed through automated capillary electrophoresis, using the ABI 3500xL Genetic Analyzer equipment (Applied Biosystems™), and the results were analyzed using the CodonCode Aligner^©^ software (CodonCode Corporation, Dedham, MA, USA) (Figure S1).

## 3. Case Report

The proband is a 25-year-old man, the only child of healthy and nonconsanguineous parents. He was born after an uneventful gestation at 39 weeks, except for discrete intrauterine bleeding at 11 weeks of gestation and the use of an unspecified intramuscular painkiller medication by the mother in the first trimester. Physical examination after delivery showed a hypotonic newborn, with the following birth measurements: length of 53 cm (+1.08 SD), weight of 4395 g (+1.70 SD), and head circumference of 37 cm (+0.68 SD). At this moment, he was also diagnosed with a cleft palate, which was surgically corrected at 12 months of age. At the age of three months, he had general hypotonia and neurological and motor development delays and started supportive therapies.

At 6 years of age, he was referred for genetic evaluation due to axial hypotonia, delayed development of fine motor skills, and behavioral anomalies (autistic behavior, restlessness, and inappropriate laughter bursts). A neuropsychological evaluation revealed the absence of speech with difficulty in global comprehension and processing (that was not related to intellectual impairment according to a specialized neuropsychological evaluation, but no IQ value was provided). He also had a history of myopia with a normal fundoscopy and recurrent episodes of otitis media, which had recovered at the time of evaluation.

**Figure 1:**
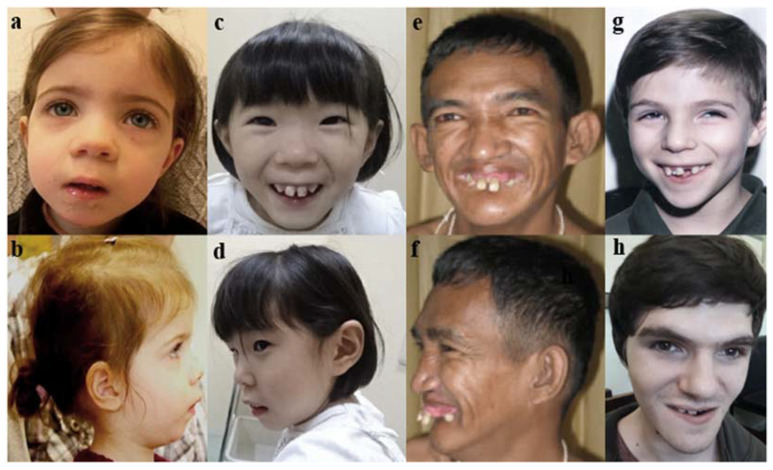
Facial appearance of individuals with c.715C>T (p.Arg239*) variant in *SATB2* ((**a**,**b**): Docker et al., 2014 [6]; (**c**,**d**): Yamada et al., 2019 [25]; (**e**,**f**): Leoyklang et al., 2007 [5]); and the proband (**g**,**h**). Note the triangular-shaped face, high anterior hairline, deep-set eyes, mild synophrys, thin vermillion of the upper lip, flat and long philtrum, dental crowding with prominent upper front incisors and prognathism.

At the physical examination, he had adequate anthropometric indexes (height: 117 cm (+0.33 SD), weight: 19 kg (−0.74 SD), and head circumference: 53.5 cm (+1.40 SD)). He had a triangular face with a high anterior hairline, deep-set eyes and prominent orbital ridges, mild synophrys, strabismus, dental anomalies (prominent incisors and dental crowding), and mandibular prognathism (Figure 1g,h). Skeletal deformities included thoracic scoliosis, pectus excavatum, lower-limb asymmetry, and valgus deformity of the right knee. The lower-limb radiographs showed valgus deformity of the left thigh with diaphysis bowing, posterior tibial bowing with slender diaphysis, and diffuse osseous decalcification and osteopenia. The serum levels of calcium and alkaline phosphatase were elevated, but the levels of parathyroid hormone (PTH) were normal. An echocardiogram revealed mitral and tricuspid regurgitation and a discrete right ventricular dilation. The renal and lower urinary tract ultrasounds were normal.

He suffered multiple bony fractures without important traumas: the left femur diaphysis at the age of 8, the left knee at the age of 9, and the left tibia at the age of 10. Because tibia X-ray images were similar to those of syphilis sabre tibia, serological tests for syphilis were performed and demonstrated a nonreagent Venereal Disease Research Laboratory (VDRL). The investigation for a secondary cause of osteoporosis was negative. At this time, in addition to daily calcium (1 g) and Vitamin D3 (1000 UI), biphosphonate (alendronate) 10 mg/day was introduced and later increased to 35 mg/dL with a significant increase in bone mineral density shown by DEXA scans. This therapy has been continuous.

At the age of 13, the patient presented aggressiveness and agitation and started using risperidone with good control. The latest physical examination was performed at the age of 25, and anthropometrical data were height: 164 cm (+1.01 SD), weight: 53 kg (+0.58 SD), adequate head circumference 57 cm (+1.88 SD), and the same dysmorphism described earlier, including facial and skeletal anomalies. He has moderate intellectual disability, but psychometric tests were not performed. Despite maintaining an absence of speech, he was able to communicate by gestures and comprehend simple commands.

Currently, the individual remains under care by a rheumatologist and neurologist, with the medication support mentioned above. He understands simple phrases and tasks, such as being asked if he is hungry or thirsty, if he wants to go for walk, or if he needs a frequently used object. He is not able to dress himself or eat without help. He attends a special school to develop his psychomotor and socialization skills, without learning how to read or write. He does not recognize colors, numbers, or letters. He lives with his parents and needs supervision for daily living care.

He still presents autistic behavior, restlessness, inappropriate laughter bursts, and aggressiveness and agitation, with a good control with risperidone. He has never presented seizures. 

During the follow-up, he was investigated by GTG-banding karyotype, fluorescence in situ hybridization with the *TUPLE1* probe, and chromosomal microarray analysis, all with normal results. At 22 years, whole-exome sequencing demonstrated a heterozygous pathogenic variant in the *SATB2* gene, a predicted loss-of-function variant *SATB2*:c.715C>T:p.(Arg239*) (GRCh37/hg19); (NM_001172509.1):c.715C>T. The segregation analysis revealed a de novo heterozygous variant c.715C>T (Figure S1).

## 4. Discussion

We present a patient with a de novo heterozygous **nonsense** variant *SATB2*:c.715C>T:p.(Arg239*) in the *SATB2* gene, predicted to cause a loss of function and consistent features of SAS, including hypotonia, developmental delay (especially absence of speech), behavioral disturbances (autistic behavior, aggressiveness, and agitation), cleft palate, dental anomalies (prominent incisors and dental crowding), progressive mandibular prognathia, recurrent episodes of otitis media, osseous decalcification, osteopenia, and osseous deformities (tibial bowing and abnormalities of the sternum), and facial dysmorphism. During subsequent evaluations, several lower-limb fractures were identified in the medical history of our case, which were probably a consequence of osteopenia and osteoporosis manifested early in life, probably due the effects of *SATB2* on bone mineralization [15,20].

The mechanism responsible for the skeletal phenotype remains unclear; however, it is known that *SATB2* plays a role in regulating skeletal development and osteoblast differentiation [20]. There is evidence that the mutations in *SATB2* may reduce pre-osteoblast proliferation [26]. A recent study showed that the *SATB2* mutations are responsible for skeletal demineralization and osteoporosis. The authors observed a high frequency of fractures and evidence of low bone mass in 19 patients carrying *SATB2* mutations. The biochemical assessment found increased alkaline phosphatase (ALP), osteocalcin, and procollagen type 1 amino-terminal propeptide (P1NP) levels, all known markers of bone formation. The increase in C-telopeptide (CTX) levels, correlated with increased bone formation markers and supports the need for bone evaluations in children and adult patients with SAS [27].

Zarate and collaborators [16] reported that nearly 70% of SAS patients presented very limited or absent speech. However, they were able to communicate to some degree by gesturing, speech-assist communication devices, and/or sign language. All these aspects are present in our patient. Another interesting point is that the present patient has never presented seizures, which is a common feature described in this syndrome.

Other findings in the patient herein described were mitral and tricuspid regurgitation and discrete right ventricle dilation. Congenital heart defects (CHDs) are a less-common finding in SAS and have been described in a few patients to date, especially in cases of large deletions involving *SATB2* and other genes [2,7,28].

In most cases, patients with large deletions have phenotypes that overlap with those of individuals affected by SAS caused by intragenic smaller deletions and/or sequence variants [1,2,7,9,10,11,16].

Eight additional individuals harboring the same pathogenic variant have been described in the literature, including another adult (Table 1). This was a 36-year-old man with craniofacial dysmorphism, cleft palate generalized osteoporosis, severe developmental delay (at this age, he only says one word), seizures, and a jovial personality [5].

In comparison with all other variants reported in Zarate et al. [16], the c.715C>T phenotype is very similar to other SAS phenotypes. Table 1 shows the main features of all individuals presenting the same variant. Figure 1 illustrates the similar facial appearances among published individuals with the c *SATB2*:c.715C>T:p.(Arg239*) variant.

SAS is not easily recognized early in life. With the increase in the assessment of next-generation sequencing in diagnostics, variants in *SATB2* have been more frequently identified. So far, there is one report discussing the natural history of SAS [28]. In this way, our case report contributes more information about the natural history of this syndrome and therefore could help with the management of SAS when diagnosed early.

## 5. Conclusions

A review of the literature shows consistency of the phenotype in SAS-related disorders, which will be more easily recognized with the increase in patients reported. This is the first report outlining the natural history of an adult male and individuals with the same variant. Focusing on the follow-up, significant limitations were observed in the autonomy of the proband, and risperidone was effective for behavior problems in our patient. Osteopenia seems to be an important symptom to be addressed.

## Figures and Tables

**Table 1:** Comparison among individuals with SAS-related disorders due to *SATB2*:c.715C>T:p.(Arg239*).

Study	Present Study	[5]	[6]	[29] Case 33	[29] Case 76	[16] Case 114	[16] Case 121	[16] Case 139	[25]	All Cases with c.715 C>T Variant	All Cases Reported ^†^
Origin	**de novo**	de novo	de novo	unknown	unknown	de novo	unknown	unknown	de novo	100% de novo (5/5)	96.7% de novo (120/124)
Age at Time of Study (years)	**25**	36	3	6.5	3	5	9.5	3	7	10.8	11
Gender	**male**	male	female	female	male	male	male	male	female	male 66.6% (6/9)	male 56.6% (86/152)
Absence of Speech	**+**	one word	+	+	one word	one word	one word	+	+	55.5% (5/9)	43.8% (64/146)
DD/ID	**+**	+	+	+	+	+	+	+	+	100% (9/9)	100% (151/151)
Cleft Palate	**+**	+	+	+	+	−	+	+	+	88.8% (8/9)	43.9% (65/148)
Feeding Difficulties	**−**	+	−	+	+	+	+	+	−	66.6% (6/9)	66.9% (83/124)
Dental Abnormalities	**+**	+	+	+	+	+	+	+	+	100% (9/9)	98.4% (129/131)
Dysmorphic Facial Features	**+**	+	+	+	+	+	+	+	+	100% (9/9)	83.4% (101/121)
Low Bone Mineral Density	**+**	+	−	+	−	NR	−	NR	−	42.8% (3/7)	70.4% (31/44)
Congenital Heart Disease	**+**	−	−	−	−	−	−	−	−	11.1% (1/9)	0% (0/152)

Legend: +: present; −: absent; NR: not reported; DD: developmental delay; ID: intellectual disability; ^†^ reported in Zarate et al., 2019 [16] excluding large deletions and the c.715 C>T variant (119 variants in 152 individuals). All percentages were adjusted to cases with data.

## Data Availability

The data generated or analyzed during this study are available from the corresponding author upon reasonable request.

## Supplementary Materials

The following supporting information can be downloaded at: https://www.mdpi.com/article/10.3390/genes14040882/s1, Figure S1. Sanger sequencing of the family.
