# Supplementary material for: SATB2-Associated Syndrome Due to a c.715C>T:p(Arg239*) Variant in Adulthood: Natural History and Literature Review
Source: Genes (Basel). 2023 Apr 8;14(4):882. doi: 10.3390/genes14040882 (PMC10137462; doi:10.3390/genes14040882)
Supplement: Supplementary file 1 [file genes-14-00882-s001.zip › genes-2333702-supplementary-updated.pdf]

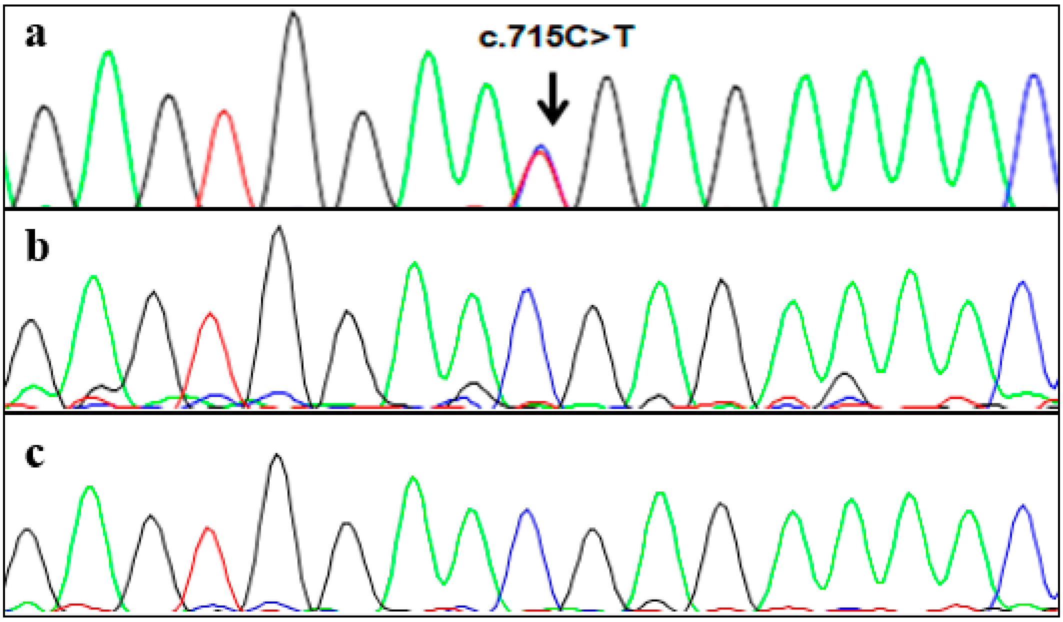

Figure S1: Sanger sequencing of the family. Electropherogram obtained from Sanger sequencing of part of exon 7 of *SATB2* gene showing the heterozygous *de novo* variant c.715C>T in proband (a), normal sequence in mother (b) and father (c).
